# Mathematical Modeling of Plant Metabolism―From Reconstruction to Prediction

**DOI:** 10.3390/metabo2030553

**Published:** 2012-09-06

**Authors:** Thomas Nägele, Wolfram Weckwerth

**Affiliations:** Department of Molecular Systems Biology, University of Vienna, Althanstraße 14, 1090 Vienna, Austria; Email: Wolfram.Weckwerth@univie.ac.at (W.W.)

**Keywords:** plant systems biology, mathematical modeling, *Arabidopsis thaliana*, natural variation, plant-environment interaction, stoichiometric modeling

## Abstract

Due to their sessile lifestyle, plants are exposed to a large set of environmental cues. In order to cope with changes in environmental conditions a multitude of complex strategies to regulate metabolism has evolved. The complexity is mainly attributed to interlaced regulatory circuits between genes, proteins and metabolites and a high degree of cellular compartmentalization. The genetic model plant *Arabidopsis thaliana* was intensely studied to characterize adaptive traits to a changing environment. The availability of genetically distinct natural populations has made it an attractive system to study plant-environment interactions. The impact on metabolism caused by changing environmental conditions can be estimated by mathematical approaches and deepens the understanding of complex biological systems. In combination with experimental high-throughput technologies this provides a promising platform to develop *in silico* models which are not only able to reproduce but also to predict metabolic phenotypes and to allow for the interpretation of plant physiological mechanisms leading to successful adaptation to a changing environment. Here, we provide an overview of mathematical approaches to analyze plant metabolism, with experimental procedures being used to validate their output, and we discuss them in the context of establishing a comprehensive understanding of plant-environment interactions.

## 1. Introduction

The performance and distribution of plants is significantly affected by several environmental factors, like for example temperature, drought and soil salinity. Due to their sessile lifestyle, plants have to cope with spontaneous or seasonal changes in these environmental factors in order to survive. To a certain degree, numerous plant species are able to respond to such changes by re-adjustment of their metabolism—a process that is in general termed as acclimation. It comprises a multitude of biochemical and physiological changes, ultimately leading to an increase in the capacity of the plants to cope with environmental stress. One prominent example, which was amongst others intensely studied in the model plant *Arabidopsis thaliana*, is the acclimation to low temperature leading to an improved cold tolerance. For many herbaceous plant species it was shown that they can grow at low temperature and even survive freezing [1,2,3]. It was demonstrated that freezing tolerance is a multigenic trait influenced by multiple factors comprising changes in gene expression, protein abundance, enzyme activity, metabolite concentrations and membrane structure [4,5,6,7,8,9]. Particularly, reprogramming of primary metabolism affects photosynthetic activity, accumulation of soluble sugars, certain amino acids and polyamines, indicating a complex relationship between metabolic consequences of low temperature. Therefore, it is not surprising that although the presence of certain sugars like e.g. sucrose or raffinose is well known to correlate with winter hardiness in many plant species [9,10], this accumulation alone is insufficient to explain the development of freezing tolerance [11]. Additionally, it is not clear whether sugars accumulate as cryoprotective substances or whether they are substrates for the synthesis of cryoprotectants, or even just as a consequence of growth retardation, which is stronger than reduction of photosynthetic activity at low temperature [12,13].

The analysis of complex metabolic processes involved in acclimation of plant metabolism to environmental stress significantly benefits from the availability of genetically distinct natural populations of the model plant *Arabidopsis thaliana*. This species is natural to Europe and central Asia, and the climate on a global scale was shown to be sufficient for shaping its range boundaries [14]. Arabidopsis has a comparatively large climatic amplitude and is spread over a latitudinal range from 68°N to 0°N, which makes it suitable for the analysis of variation in adaptive traits [15,16]. Due to being a predominantly selfing species, most individual Arabidopsis plants collected in nature are homozygous inbred lines and are commonly referred to as accessions which are specialized to particular sets of environmental conditions. The usefulness of accessions in exploring plant cold acclimation mechanisms was exemplified by Hannah and co-workers who demonstrated that the freezing tolerance of nine natural accessions, originating from Scandinavia to the Cape Verde Islands, correlates with habitat winter temperatures [6]. Additionally, they showed that low temperature represents an important selective pressure for Arabidopsis. 

Besides such complex plant-environment interactions, latest developments in bioanalytical research comprising shotgun and next-generation genome sequencing as well as molecular analysis using OMICS technologies have driven the need for computer-assisted analysis and modeling of biological data. Systems biology has evolved in a research field focusing on the system wide understanding of biological networks, like for example the cellular metabolism in a photosynthetically active plant cell. In a systems biology approach, network elements, such as genes, proteins or metabolites, are considered as interacting components rather than isolated entities in order to deepen the comprehensive understanding of the organization of a complex biological system. A promising way to analyze such complex biological and biochemical networks is formal representation by mathematical models enabling their computer based handling and making biological data accessible to theoretical methods originating from applied mathematics and systems theory. Numerous mathematical approaches to model plant metabolic networks have been suggested and discussed, both relying on and emphasizing the importance of the iterative processes of model development, simulation and validation by experimental data [17,18,19,20,21,22]. An overview of several computational approaches to study metabolic networks with respect to features like topology, stability and dynamical behavior was provided previously [23], classifying the approaches by their mathematical structure. Thus, qualitative models for a static network description containing no kinetic parameters (network/stoichiometric analysis) were distinguished from quantitative approaches applying a dynamic system description using kinetic parameters (kinetic models) [23].

A main focus of mathematical modeling in biochemistry and plant science has been on the construction of kinetic models where metabolic states are simulated based on the knowledge about network topology, stoichiometry, rate equations and kinetic parameters. Typically, a system of ordinary differential equations (ODEs) is used to describe the time-dependent changes in state variables, *i.e.,* metabolite concentration, protein abundance or the amount of transcripts. In the context of metabolic systems, the sum of synthesizing and degrading rates of enzyme reactions defines the time-dependent change in metabolite concentrations. The representation of biological systems by sets of ODEs has successfully been applied to various processes in plant biology and also in the comprehensive analysis of plant-environment interactions, as was already outlined in [24]. While kinetic modeling represents an attractive method to study and beyond that, to potentially predict the behavior of complex metabolic networks, plenty of information about the network topology and the kinetics of metabolite interconverting steps is required for model development and its experimental validation [25]. For example, although rates of enzymatic steps can in general be reliably approximated by Michaelis-Menten kinetics, in many cases a probable influence of allosteric effectors is not confirmed but may have a dramatic effect on systems dynamics, and negligence will have a significant impact on the model output, *i.e.*, the result of model simulation [26]. Additionally, in many cases mechanisms of allosteric regulation are known but quantitative experiments on parameters like substrate affinity (K_M_) or inhibitory constants (K_i_) are lacking. This enforces the application of parameter estimation to calculate parameter values which are either completely unknown or can be estimated within numerical bounds based on published data on a different condition or organism. Indeed, such assumptions cause uncertainties, which have to be discussed carefully when interpreting the model output. However, although there might be several uncertainties with respect to regulatory instances involved in every single reaction of metabolism, numerous studies have proven kinetic modeling to be a promising approach to comprehensively analyze complex processes in plant biology. An overview of applications is given by Schallau and Junker [27] exemplarily comprising the process of photosynthesis [28], leaf carbon metabolism [29], sucrose metabolism in sugar cane (*Saccharum officinarum*) [30] or the aspartic acid-derived amino acid pathway in *Arabidopsis thaliana* [31].

In contrast to kinetic modeling, the approach of structural modeling is based on the idea of constructing models without kinetic information. This modeling approach refers only to the stoichiometry of the reactions within the system which is summarized in the stoichiometric matrix **N**. Considering a metabolic reaction network, each column of **N** represents a reaction while rows represent metabolites. Hence, the elements of **N** describe the stoichiometric coefficients of metabolites in the reactions. Positive entries indicate that the metabolite is produced by the reaction, while negative values indicate consumption. Entries of zero indicate that the metabolite is not involved in this reaction. The definition of a vector *v* containing the rates of metabolite interconversions allows for the description of the steady state of the metabolic reaction network by a set of differential equations:


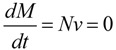
(1)

where **M** represents a matrix containing metabolite concentrations and **t** is time. Solutions of this equation can be calculated applying linear algebraic rules. The advantage of this approach becomes obvious when considering the large number of reactions in a metabolic system, which can be predicted from an annotated genome sequence. The workflow from metabolic reconstruction to modeling of a metabolic network based on an annotated genome sequence was previously described in detail [32]. Sequence data of a genome are derived from techniques like next-generation sequencing, which are capable of sequencing a whole prokaryotic or eukaryotic genome within days. The assembled complete genome sequence is analyzed for intron/exon structure, start and stop codons as well as homology with known sequences. A genome-scale functional annotation based on the homology with functionally characterized genes from other organisms results in a gene list, which is used to predict enzymatic reactions. Based on the substrates and products of the enzymatic reactions, pathways are structured and a stoichiometric matrix can be derived. Applying this approach, it becomes possible to analyze a large set of metabolic interactions simultaneously, and results of experimental high-throughput studies on the metabolome and proteome are a promising way to validate the model output in metaproteogenomic studies as demonstrated for *Chlamydomonas reinhardtii* [33]. Yet, in context of metabolism of higher plants the main challenges of this modeling approach result from the genome content whose function remains undiscovered, and also from characteristics like subcellular compartmentation and tissue differentiation making the analysis of higher plants much more complex than in prokaryotic organisms [34]. 

Focusing now again on the complexity of plant-environment interactions and the variability of stress responses in natural accessions of *Arabidopsis thaliana*, both kinetic and stoichiometric modeling represent promising approaches to comprehensively study regulatory instances in plant metabolism, its re-adjustment after environmental perturbations or even the impact of changes in transcriptional control on the metabolome. On the other hand, both applications of mathematical modeling are significantly limited in their ability to reconstruct and predict the behavior of plant metabolism *in vivo*. Kinetic modeling typically focuses on a relatively small part of metabolism and aims at simplification to constrain the complexity and amount of kinetic information, which is needed to simulate network dynamics. In contrast, approaches of stoichiometric modeling focus on the comprehensive compilation of network interaction, ultimately aiming at the complete representation of metabolism, yet neglecting kinetic information and the estimation of non-linear network dynamics. Motivated by the limitations of each of these methods, the following sections are intended to summarize current progress in mathematical modeling of plant metabolism and to figure out its potential to analyze and predict complex plant-environment interactions. 

## 2. Estimating Dynamics of Plant Metabolism Due to Environmental Perturbation

The mathematical analysis of plant metabolism first of all relies on the representation by a model, which is constructed based on information on biochemical pathways and the interaction of pathway components gained from numerous previous experimental studies. Typically, the first step of model construction consists of a graphical representation of the pathway or network of interest. In case of a kinetic model of metabolism, this may result in a map, which connects nodes by lines. Every node now represents a metabolite and every line describes a metabolite interconversion, *i.e.*, enzymatic reaction. Based on this graphical representation, the mathematical model is then derived by translating nodes and lines in metabolite concentrations and enzymatic rate laws, for example the Michaelis-Menten equation. These rate equations are characterized by kinetic parameters like enzymatic substrate affinity, *i.e.*, the Michaelis-Menten constant K_M_, and the maximum enzyme activity v_max_. This process is crucial for the successful modeling approach as all further steps of mathematical analysis rely on these assumptions: if the interaction between two network components is described by equations or parameters which do not agree with confirmed experimental results, validation of simulation results by experimental data is not reliable anymore and the model becomes unfeasible. Although a vast number of metabolic interactions have intensively been characterized and many underlying laws of interaction are well known, like for example the Michaelis-Menten kinetics, deriving the most realistic model structure of a metabolic network becomes difficult when assumptions about simplification have to be made. This is frequently the case for kinetic models, based on systems of ODEs, which are intended to provide an insight into the dynamics of metabolism. These dynamics are predominantly nonlinear and model systems are often characterized by a high-dimensional parameter space. Kinetic parameters, characterizing substrate affinity (K_M_) or inhibition (K_i_), are often not directly accessible to experimental measurements. In addition to the everlasting question how results of *in vitro* measurements differ from *in vivo* data, experimental conditions, like the temperature or pH, significantly constrain their validity. Hence, besides the determination of a model structure, the process of mathematical identification of unknown kinetic parameters represents another crucial step in building a realistic ODE-based model to simulate dynamics of plant metabolism. To reduce the complexity and also the number of unknown kinetic parameters, individual enzymatic steps might be summarized in blocks of interconversions directly linking the metabolite concentrations that have been quantified. These blocks of interconversion are confined by the rate-limiting steps, *i.e.*, the enzymatic reaction representing a regulatory bottleneck for the synthesis/degradation of a metabolite. Measurement on the kinetic parameters of the corresponding enzymes then allows for the estimation of the kinetic characteristics of this metabolic pathway. This approach was recently applied to the analysis of diurnal dynamics of the central carbohydrate metabolism in leaves of *Arabidopsis thaliana* [35]. For modeling, the authors used a simplified scheme of central carbohydrate metabolism, focusing on the most abundant sugars and phosphorylated intermediates at which pathways branch. In this simplified model, subcellular compartmented pathways were lumped together and aerial organs of the vegetative plant were collectively interpreted as a source of carbon fixation that exports carbon to sink organs, for example roots [35]. The modeling approach was applied to wild type plants as well as mutant plants defective in the dominating vacuolar invertase AtβFruct4 (At1G12240) to analyze the physiological role of AtβFruct4 on whole plant carbon metabolism. Although this approach is based on assumptions significantly simplifying the *in planta* metabolic network, the authors were successful in reproducing the experimentally observed data on metabolite concentrations. Furthermore, simulation results allowed for the evaluation of flux rates in the central carbohydrate metabolism revealing a significant impact of invertase activity on sink-source interaction and buffering metabolite concentrations against changes in environmental conditions [35]. The usefulness of this modeling approach to study dynamics of metabolism induced by environmental perturbations was underpinned by application to the analysis of the regulation of the central carbohydrate metabolism during cold acclimation [22]. In this study, the authors applied an extended version of the mathematical model presented in [35] to analyze diurnal dynamics in carbohydrate metabolism of natural accessions of *Arabidopsis thaliana*, now also comprising the central steps of raffinose interconversion. Based on results of model simulation, a critical temperature for sucrose synthesis in a cold sensitive accession could be predicted at which an imbalance in photosynthetic carbon fixation is caused, ultimately resulting in oxidative stress [22]. It was concluded that metabolic capacities contribute to the ability of accessions of *Arabidopsis thaliana* to cope with changes in environmental conditions at low temperature. 

As exemplified by these approaches of mathematical modeling, realistic output of model simulations can be expected despite a significant simplification of the model structure. This was also proven by many other kinetic modeling approaches (for an overview of several approaches please refers to [20]). Such simplifications may be performed in order to reduce the number of unknown model parameters and to minimize ambiguity of the model output. This ambiguity occurs due to uncertainties concerning model parameters as well as experimental data, kinetics and model structure. These different types of uncertainty are interlaced because uncertain network structures contain uncertain reaction kinetics that are characterized by uncertain parameters [36]. Assuming that both the model structure and kinetic laws are known, then parameter values have to be estimated allowing for the simulation of experimental data, for example metabolite concentrations. While uncertainty of unknown or marginally characterized parameters will be huge, extensive experiments on parameters will significantly contribute to unambiguous simulation results. Additionally, experimental data on dynamics of the model components, like for example time courses of metabolites, will contribute to minimization of parameter uncertainty: only parameter sets allowing for the successful simulation of the time-course will be approved. The parameter estimation of nonlinear dynamic modeling approaches can be classified as a nonlinear programming problem being subject to nonlinear differential-algebraic constraints [37]. In general, this mathematical problem can be formulated as follows:


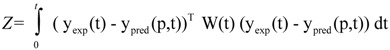
(2)

where Z represents the cost function to be minimized, y_exp_ contains experimentally determined state variables (for example metabolite concentrations), y_pred_(p,t) is the model prediction of state variables depending on estimated parameters p and time t, and W(t) is the weighting matrix containing information about the level of importance of single state variables and determining their influence on the cost function. This optimization problem of minimization of Z is subject to the differential/algebraic equality constraints describing the systems dynamics and additional requirements for system performance. Additionally, the estimation of model parameter p is subject to lower (p^low^) and upper (p^up^) bounds:


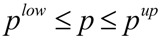
(3)

Due to nonlinearities in objective function and constraints, solving these optimization problems frequently means having to cope with multimodality, *i.e.*, the potential existence of multiple local solutions [37,38]. This implies the application of algorithms, which are able to overcome local minima to ultimately yield the best solution, *i.e.*, the global optimum. Gradient-based local optimization methods fail to reliably determine the global optimum in multimodal problems because of nonconvexity arising from the previously mentioned nonlinearities. A graphical representation of this problem is shown in [38]. In a simple example it was demonstrated that even with only two decision variables, e.g., unknown kinetic model parameters, multimodal surfaces may result from optimization problems, *i.e.*, surfaces of the cost function with multiple peaks and valleys, which do not allow for the determination of one unique optimal solution by local optimization methods. Solving such multimodal problems is the goal of global optimization [39], which was discussed and reviewed in the context of parameter estimation in biochemical pathways [37]. One example for a global optimization method is the particle swarm pattern search method for bound constrained global optimization [40]. This algorithm was shown to be highly competitive with other global optimization methods and is a demonstrative example of how possible nonconvexity of the objective function can be globally explored. The basic idea behind this approach is to construct a hybrid of a pattern search method and a particle swarm search [40]. A particle swarm algorithm attempts to simulate the social behavior of a population of particles to explore a given problem space [41]. For every iteration step of the optimization process, the particles are associated with a stochastic velocity vector indicating the particles’ direction of movement. The velocity vector for each particle is a linear stochastic combination of the velocity at the previous time instant, of the direction to the particle’s best position, and of the direction to the best swarm position. The new position of every particle is calculated by adding the current velocity vector to the old particle position. The stopping criterion for the algorithm may then be defined by a tolerance level of velocities, which has to be reached for all particles. While pattern search methods are designed to achieve convergence from arbitrary starting points to points satisfying necessary conditions for local optimality [42], the incorporation of a particle swarm search in the search step of a pattern search method enables the attraction of local optima and the identification of global optima to be overcome [40].

Due to its capability to develop methods for comprehensive analysis of complex data sets and provide strategies of how to solve nonlinear problems, optimization theory represents an essential component for mathematical modeling of plant metabolism and other biological systems. Beyond that, the prediction of metabolism from first principles only becomes possible by application of optimization approaches [43].

## 3. Modeling on a Large Scale—Reconstruction of Metabolic Networks and Validation of Predictions by Metabolomics Science

Reconstruction of metabolic networks is based on information about whole genome sequences finally resulting in the stoichiometric matrix **N **of the network, which provides the basis for all modeling approaches [32]. As described in the previous section, particularly in kinetic modeling approaches, this information is frequently reduced in order to minimize complexity and unambiguous model outputs. In contrast, stoichiometric modeling approaches aim at the compilation and integration of the entire stoichiometric information of the metabolic network. Numerous missing enzyme parameters prevent comprehensive analysis by kinetic modeling, yet determination of steady-state solutions for the metabolic network is possible by solving equation (1) numerically. Compared to the complex analysis of nonlinear dynamical systems, this system of linear equations can easily be solved. However, the complexity of such an approach is indicated by the comprehensive reconstruction process as well as the experimental validation, revealing the need for permanent improvement of published metabolic network reconstructions by biochemist experts’ knowledge and proteogenomic methods [33,44,45]. In a detailed protocol, Thiele and Palsson described the complex reconstruction process within four major steps leading to a metabolic network model [46]. Based on an automated draft reconstruction, the network model is iteratively refined, converted into a computable format and evaluated by the comparison of model predictions with experimental results on physiology, biochemistry or genetics. Although the process of reconstruction is identical for prokaryotic and eukaryotic metabolic networks, the authors emphasize that in eukaryotic systems, e.g., metabolism of higher plants, it is more challenging due to the size of genomes and cellular compartmentation [46]. Additional complexity arises from network gaps and mass-balance errors resulting from incomplete genome annotation and reaction stoichiometry errors which severely affect the predictive power of network models [47]. Beyond that, model simulations provide only information about a steady state, *i.e.*, a snapshot, of the system, which is pre-defined by the experimental design. Recently, in several studies genome-scale metabolic modeling in *Arabidopsis thaliana* was applied to address questions like ATP demand for growth and maintenance [21], the metabolic activity of key enzymes responsible for the supply of redox equivalents in plastids during the photorespiratory cycle [48] or to predict the design of genetic manipulations that are expected to increase vitamin E content in metabolically engineered seed strains [49]. 

With respect to such comprehensive metabolic network simulations, quantitative measurement of metabolism is necessary to validate the output of such simulations, which can be accomplished applying bioanalytical methods in metabolomics science [50]. Mass spectrometry is one of the crucial technologies in this field, and an overview of different techniques in context with their characteristic features has recently been presented [32]. A recent development is the use of two-dimensional gas chromatography coupled with fast acquisition rate time-of-flight mass spectrometry (GC x GC-TOF-MS). The coupling of two gas chromatography columns with different characteristics, for example a hydrophobic and a polar column, increases the separation efficiency of a complex metabolomics sample. A complete strategy to perform a convenient data extraction and alignment using two-dimensional gas chromatography coupled with mass spectrometry (GC x GC-MS) technology is already available [51]. Another important extension of current metabolomics platforms for metabolomics is the integration of gas chromatography coupled to mass spectrometry (GC-MS) with liquid chromatography coupled to mass spectrometry (LC-MS) [52]. This approach enables the analysis of components of the primary metabolism by GC-MS, for example carbohydrates and amino acids, and higher molecular masses by LC-MS, e.g., secondary metabolites [53,54]. Beyond the development of techniques and new platforms, the improvement of databases, experimental standards and data compatibility among different laboratories is crucial for efficient metabolomics science [55]. 

The analysis of metabolomics results on numerous biological replicates under different environmental conditions or with genotypic variation represents a multidimensional task and results in a complex data matrix. The covariance matrix C results from multivariate statistics representing a central result of the experiments [32,56,57,58,59]. The observed covariance matrix C of metabolite concentrations is linked to the underlying biochemical system and the corresponding genotype by a systematic approach, which is characterized by the following equation [60]:


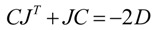
(4)

In this equation, J represents the Jacobian matrix and D is the fluctuation/diffusion matrix. The diagonal entries D_ii_ characterize the magnitude of fluctuations of each metabolite, whereas off-diagonal entries D_ij_ (i≠j) represent the fluctuation of metabolites caused by the interaction between enzymes i and j. The interconnection between metabolic networks and the Jacobian Matrix as well as the fluctuation matrix is described in detail elsewhere [32,60,61]. In general, the Jacobian matrix characterizes the local dynamics at a steady state condition. In the context of metabolic networks, the entries of the Jacobian J represent the elasticities of reaction rates to any change of the metabolite concentrations being characterized by the following equation:


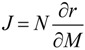
(5)

Here, N is the stoichiometric matrix, r represents the rates for each reaction and M is the metabolite concentration. Based on equations (4) and (5), an approach of inverse calculation of a Jacobian from metabolomics covariance data was recently derived [59]. Additionally, the authors developed the differential Jacobian, dJ_ij_, defining the relative change of two Jacobians J_a_ and J_b_ which are associated with different treatments, *i.e.*, environmental conditions:


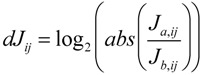
(6)

Calculation of the differential Jacobian reveals perturbation sites between two different metabolic states hinting at a significant regulatory event, e.g., the change of enzymatic reaction rates due to environmental perturbations. In principle, using this approach it is possible to conveniently connect a large metabolomics experiment with many samples and thousands of variables directly with the predicted genome-scale metabolic network to calculate biochemical regulation in the investigated biological system (for more detail see [32]). The approach relies on the assumption that regulation of metabolism becomes observable in the significant changes of the local dynamics around a steady state condition, e.g., rates of metabolite synthesis and degradation. Due to the redundancy of pathways and multiple isoforms of numerous enzymes, such calculations and predictions need to be confirmed and validated by further biochemical experiments. Limitations to this approach are currently the low quality knowledge of N and the low number of detected metabolites in measurements compared to the number of predicted metabolites in a metabolome, necessitating the simplification of N in accordance with the data matrix [32,59]. 

## 4. Conclusions

**Figure 1:**
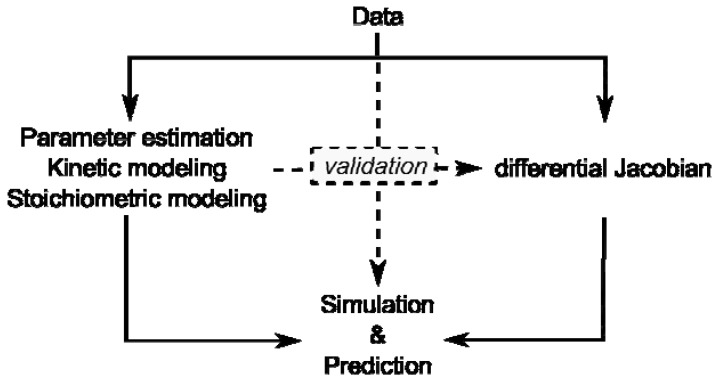
Overview of modeling approaches and their interaction by validation. Data represent results of experiments on the metabolome, proteome, enzyme activities or transcriptome.

With regard to currently emerging discussions about world population feeding, global climate change and limited energy resources with fossil fuels, plant biology and biotechnology are central topics of life sciences in the coming decades [53]. Latest developments in bioanalytical research aim at the understanding of organisms at a systems level and within their ecosystemic context. Characterized by nonlinearities and multidimensionality, the comprehensive analysis of plant-environment interactions is non-intuitive. Thus, the application of methods, which are capable of coping with this complexity, is necessary. Mathematical modeling and computer-assisted data analysis are powerful and adequate approaches used to exploit entire data sets provided by experimental high-throughput technologies in order to derive a new hypothesis about regulation of biological systems. Although every single mathematical approach is limited by underlying assumptions, the combination of different modeling approaches may yield the ultimate amount of information available from experimental data sets (Figure 1).

Kinetic modeling approaches are limited by lack of kinetic information and stoichiometric modeling approaches are limited by their reference to a steady state. Yet, if a stoichiometric modeling approach delivers information about potential perturbation sites in metabolism, this will enable systematic in-depth analysis, for example by kinetic modeling, promoting a comprehensive understanding of how plant metabolism is composed functionally.
